# Archimedes Optimization Algorithm with Deep Learning-Based Prostate Cancer Classification on Magnetic Resonance Imaging

**DOI:** 10.3390/healthcare11040590

**Published:** 2023-02-16

**Authors:** Mahmoud Ragab, Faris Kateb, E. K. El-Sawy, Sami Saeed Binyamin, Mohammed W. Al-Rabia, Rasha A. Mansouri

**Affiliations:** 1 Information Technology Department, Faculty of Computing and Information Technology, King Abdulaziz University, Jeddah 21589, Saudi Arabia; 2 Department of Mathematics, Faculty of Science, Al-Azhar University, Cairo 11884, Egypt; 3 Faculty of Earth Sciences, King Abdulaziz University, Jeddah 21589, Saudi Arabia; 4 Geology Department, Faculty of Science, Al-Azhar University (Assiut branch), Assiut 71524, Egypt; 5 Computer and Information Technology Department, The Applied College, King Abdulaziz University, Jeddah 21589, Saudi Arabia; 6 Department of Medical Microbiology and Parasitolog, Faculty of Medicine, King Abdulaziz University, Jeddah 21589, Saudi Arabia; 7 Health Promotion Center, King Abdulaziz University, Jeddah 21589, Saudi Arabia; 8 Prince Sattam Bin Abdulaziz University, Al-Kharj 11942, Saudi Arabia; 9 Department of Biochemistry, Faculty of Sciences, King Abdulaziz University, Jeddah 21589, Saudi Arabia

**Keywords:** artificial intelligence, healthcare, prostate cancer, medical imaging, deep learning

## Abstract

Prostate cancer (PCa) is becoming one of the most frequently occurring cancers among men and causes an even greater number of deaths. Due to the complexity of tumor masses, radiologists find it difficult to identify PCa accurately. Over the years, several PCa-detecting methods have been formulated, but these methods cannot identify cancer efficiently. Artificial Intelligence (AI) has both information technologies that simulate natural or biological phenomena and human intelligence in addressing issues. AI technologies have been broadly implemented in the healthcare domain, including 3D printing, disease diagnosis, health monitoring, hospital scheduling, clinical decision support, classification and prediction, and medical data analysis. These applications significantly boost the cost-effectiveness and accuracy of healthcare services. This article introduces an Archimedes Optimization Algorithm with Deep Learning-based Prostate Cancer Classification (AOADLB-P2C) model on MRI images. The presented AOADLB-P2C model examines MRI images for the identification of PCa. To accomplish this, the AOADLB-P2C model performs pre-processing in two stages: adaptive median filtering (AMF)-based noise removal and contrast enhancement. Additionally, the presented AOADLB-P2C model extracts features via a densely connected network (DenseNet-161) model with a root-mean-square propagation (RMSProp) optimizer. Finally, the presented AOADLB-P2C model classifies PCa using the AOA with a least-squares support vector machine (LS-SVM) method. The simulation values of the presented AOADLB-P2C model are tested using a benchmark MRI dataset. The comparative experimental results demonstrate the improvements of the AOADLB-P2C model over other recent approaches.

## 1. Introduction

Prostate cancer (PCa) is a major factor for the increasing death rates in cancer and is most commonly identified among men [1]. In spite of its prevalence, PCa can be frequently non-aggressive, making it difficult to find. This results in the disease presenting higher risks to patients as to warrant treatments, such as radiation therapy or prostatectomies (prostate surgery) [2]. Histopathologically, Gleason grading can be a robust prognostic predictor in prostate carcinoma [3], but Gleason grading is difficult to execute and subjective, with important intra- and inter-observer variabilities. Whereas uropathologists have a higher agreement rate, this skill is often unavailable. The current guidelines require the involvement of pathologists in determining the proportion of cancer through various Gleason grading systems, which increases the workload for pathologists and exacerbates subjective issues [4]. In recent decades, research has shifted toward utilizing Artificial Intelligence (AI) and statistics to improve the accuracy of estimation and diagnosis outcomes. The utility of computer-oriented learning techniques is becoming the main research area in PCa [5], and Artificial Neural Networks (ANN) are progressively being utilized for building advanced prognostic methods for the detection of PCa. To obtain structured data which include outcomes and input variables with some knowledge of PCa insights, it is enough to train a machine learning model [6]. For example, numerous new tools are available for diagnosing and screening PCa, such as biomarkers (molecular imaging and exosomes), genomics, and magnetic resonance imaging (MRI). In this case, AI has an important role in two ways. By analyzing large amounts of data and leveraging advancements in machine learning (ML) techniques, urologists can reduce the number of unnecessary prostate biopsies while maintaining the accuracy of identifying aggressive prostate cancer [7].

Furthermore, the usage of AI, extracellular vehicles, and genomics may offer a rapid and more reliable PCa test. Machine learning (ML) is a subfield of Artificial Intelligence that involves the development and deployment of techniques for analyzing data and its characteristics. It does not require specific inputs from the environment to perform its tasks [8]. ML methods are categorized in accordance with the kind of feature and label. For labeling, ML is categorized into three methods, including reinforcement learning, supervised, and unsupervised methods. Regarding features, ML is categorized into non-handcrafted or handcrafted feature-oriented approaches [9,10,11,12]. Deep Learning (DL) is a kind of ML that allows machine devices to learn from experiences and realize atmosphere with regard to a hierarchical model. Computers gain knowledge through learning experiences, and there is no need for a human to provide every piece of information beforehand [13]. Currently, the Deep Convolutional Neural Network (DCNN), an altered form of Artificial Neural Networks (ANN), has been proven to have high efficacy if implemented to digitalize images, a type of computer-aided diagnosis (CAD) analysis.

This article applies an Archimedes Optimization Algorithm with Deep Learning-based Prostate Cancer Classification (AOADLB-P2C) model to MRI images. The presented AOADLB-P2C model majorly examines MRI images for the identification of PCa. To accomplish this, the AOADLB-P2C model performs pre-processing in two stages: adaptive median filtering (AMF)-based noise removal and contrast enhancement. Furthermore, the presented AOADLB-P2C model extracts features via a densely connected network (DenseNet-161) model with a root-mean-square propagation (RMSProp) optimizer. Finally, the presented AOADLB-P2C model classifies PCa using the AOA with a least-squares support vector machine (LS-SVM) method. The simulation values of the presented AOADLB-P2C model are tested using a benchmark MRI dataset. In short, the key contributions of the current study are listed below:

- An intelligent AOADLB-P2C technique is presented, and it comprises AMF-based pre-processing, DenseNet-161-based feature extraction, RMSProp optimizer, LS-SVM classification, and AOA-based hyperparameter tuning. To the best of the researchers’ knowledge, a AOADLB-P2C model has never been presented in the literature.
- A RMSProp optimizer is applied in this study for the selection of hyperparameters involved in the DenseNet-161 model.
- The parameter optimization of the LS-SVM model using the AOA algorithm and cross-validation helps in boosting the predictive outcome of the proposed model for unseen data.
- The performance of the proposed model is validated using a PCa dataset.

The rest of the paper is organized as follows. Section 2 provides a detailed review of the literature and Section 3 introduces the proposed model. Next, Section 4 offers the comprehensive analytical results and Section 5 concludes the work.

## 2. Related Works

The current section provides a detailed survey of existing models related to PCa classification. Liu et al. [14] modeled a DL method by combining the Inception-v3 and S-Mask region-based Convolutional Neural Network (R-CNN) models and used it in the ultrasound image-aided prognosis of PCa. The enhanced S-Mask R-CNN was leveraged to achieve a precise segmentation of the generated candidate regions and prostate ultrasound images. Furthermore, the RoI align method was also leveraged to realize the pixel-level feature point position. The respective binary mask of the prostate images was produced by the convolutional networks to distinguish the background and the prostate regions. In [15], a new automated classifier technique was modeled by merging several DL techniques to identify PCa from MRI and ultrasound (US) images. The devised technique also explains why a particular decision is given for the input MRI or US image. Numerous pretrained DL methods with custom-developed layers were included in this study on top of the corresponding pre-trained techniques and were implemented in the database.

Toledo-Cortés et al. [16] modeled a quantum-inspired deep probabilistic learning ordinal regression approach for medical image diagnosis. The proposed model leverages the advantages of intrinsic ordinal information of disease phases and representational power of DL techniques. The technique was assessed using two distinct medical image analyzing tasks, including the diabetic retinopathy grade estimation on eye fundus images and PCa diagnosis. Alam et al. [17] devised and authenticated several classifier methods on a supervised ML algorithm to forecast the occurrence of PCa. A modified LR method was modeled and applied on the images captured from patients who are vulnerable to PCa. This devised classifier method used various stages of tumor and clinical features. The clinical features included smoking history, BMI, cystitis infections, and age. Zhong et al. [18] conducted research work in which the main objective was to devise a Deep Transfer Learning (DTL)-oriented method for differentiating indolent lesions from clinically significant (CS) PCa lesions. The researchers compared the DTL-related method with a DL technique without TL and PIRADS v2 score over three Tesla multi-parametric MRI (3T mp-MRI) with Whole Mount Histopathology (WMHP) validations.

Wang and Wang [19] formulated a DL-oriented technique for automatic classification of clinically significant (CS) and clinically insignificant (CiS) PCa based on multiparametric MRI (mpMRI) images. Their study also intended to select suitable mpMRI series for PCa categorization in various anatomic zones. For the selection of optimal integration of the series for PCa classification in a particular zone and PCa classification, the researchers devised Multi-Input Selection Networks (MISNs). A MISN is a multi-input or multi-output classifier network that has a total of nine branches for processing nine input images from the mpMRI data. Poojitha and Sharma [20] discussed the saliency maps of images utilizing Deep Convolutional Generative Adversarial Networks (DCGANs) by implementing a semantic segmentation method with salient maps, offered by pathology specialists. This structure was modeled by integrated the fine-tuned VGGnet, CNNs, and RNNs. The authors presented a new method in which LSTM-RNN was used for sequential sub-band images of shearlet coefficients.

According to [21], novel urinary and serum biomarkers have been established in recent years. However, the researchers continuously search for novel biomarkers under different conditions and patient settings. In spite of these, there is a lack of particular rules with high level of evidence on the utilization of these markers. The count of the analysis, which focuses on the characterization of a particular PCa metabolic phenotype by utilizing distinct experimental methods, has been reported. In a study conducted earlier [22], a dual 5α-reductase inhibitor (5-ARI), i.e., dutasteride, was found, and it blocks testosterone from being converted into its active element, i.e., dihydrotestosterone (DHT), and decreases prostate volume, thus enhancing urinary flow rate. The Bipolar Transurethral Resection of the Prostate (B-TURP) process tends to enhance the outcomes and efficiency in comparison with the typical TURP, yet the incidence of side-effects in the B-TURP is lower.

Ferro et al. [23] reviewed existing evidence on the approaches and examinations conducted in existing literature on radiomics in PCa patients. The review also recommends its potential in terms of personalized medicine and future applications. The analysis conducted by Massanova et al. [24] related several means of PV estimate, comprising Digital Rectal Examination (DRE), Transrectal Ultrasound (TRUS), MRI, and radical prostatectomy specimens to determine the best method for volume measurement.

Several CAD models have been proposed in the literature for PCa classification process. Although several ML and DL models are available in the literature for liver cancer classification, there is a need to enhance the classification performance. Owing to the continuous exploration of this research domain, the number of parameters in DL models also increases quickly, which in turn results in model overfitting. At the same time, different hyperparameters exert a significant impact on the efficiency of CNN models. Particularly, hyperparameters, such as epoch count, batch size, and learning rate selection, are essential to attain an effectual outcome. Since the ‘trial and error’ method for hyperparameter tuning is a tedious and erroneous process, metaheuristic algorithms can be applied. Therefore, the current study employs the RMSProp optimizer for DenseNet and AOA algorithms in a LS-SVM model.

## 3. The Proposed Model

**Figure 1:**
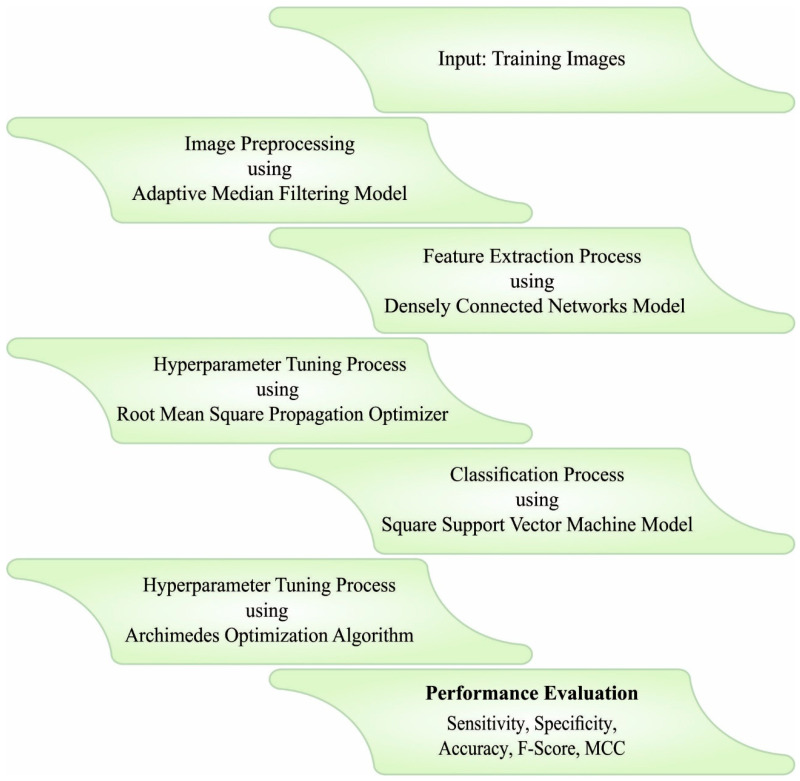
Overall working process of the AOADLB-P2C system.

In this article, the authors propose a novel AOADLB-P2C method for PCa diagnosis from MRI images. Initially, the proposed AOADLB-P2C model pre-processes the MRI images in two stages, i.e., AMF-based noise removal and contrast enhancement. Moreover, the RMSProp optimizer with the DenseNet-161 model is applied for the purpose of feature extraction. Finally, the presented AOADLB-P2C model classifies the images for PCa using the AOA with LS-SVM models. Figure 1 shows the overall working process of the proposed AOADLB-P2C system.

### 3.1. Pre-Processing

At first, the proposed AOADLB-P2C model pre-processes the MRI images in two stages using AMF-based noise removal and contrast enhancement [25]. A simple MF is employed at the median of the window to replace the centre pixel that is regarded as a window. Once the central pixel is categorized as (salt) or (pepper), it is replaced by the middle value of the window. The major drawback of the standard MF is that, although the pixels are regarded as incorrect (except 0 or 255), it gets substituted with the median of the window. This reduces the whole visual quality of the image. In addition, it is not possible for an MF to preserve the edges. In general, the window is sorted in an ascending order. The median is deemed to be the middle value. Therefore, the undamaged pixels get substituted with the median value. The following stage is to employ the Contrast Limited Adaptive Histogram Equalization (CLAHE) approach on the noise-removed images. It has the best tractability in electing the local histogram map ping function. The CLAHE approach separates the images into suitable regions and enforces the histogram equalization approach upon them. Then, the clipped pixels are reassigned to every gray level. This novel histogram is unlike the typical histogram since the intensity of all the pixels gets constrained by the user-selectable maximum number. Hence, the CLAHE model might limit the noise enhancement outcome.

### 3.2. Feature Extraction

At this stage, the RMSProp optimizer is applied with the DenseNet-161 model for the purpose of feature extraction [26]. This model possesses dense convolution network (DenseNet) that accomplishes the maximum classification performance on large data, such as CIFAR-10 and ImageNet. The ResNet model acquires a duplicate feature map and contains several parameters, which make the training process a difficult one. On the other hand, the DenseNet model is comprised of thin layers and learns from a small number of feature maps, whereby every layer feeds data to the adjacent layer. The connecting model has more than one convolutional layer in a dense block of the DenseNet model. This feed-forward connection raises the overall number of layers from L to L(L+1)/2. Consequently, the network gets trained in an effective manner, which not only reduces the over-fitting issue but also enhances the performance of the model. The DenseNet-161 model correspondingly comprises four dense blocks with (6, 12, 36, and 24) sub-blocks. Every sub-block has two convolutional layers that result in a total count of 156 layers with 5 convolutional layers having a growth rate of k=48. Here, k represents the feature map. In model compression, the transition layer plays a crucial role. If the number of channels in the output feature maps of the dense block is m, then the subsequent transition layer creates [θm] output feature map. Here, 0<θ≤1 represents the compression factor. When θ=1, the number of feature mappings through the transition layer remains unchanged. This model is appropriate for embedded devices due to its thick compressibility and connectivity. With the help of the DenseNet-161 model as the pre-trained module, the knowledge (weight value) of the basic structure learnt in the first and middle layers gets transmitted to the presented method. The fundamental parameter, i.e., the pre-trained model, learns to categorize different objects in the ImageNet, and it has been utilized for the classification of the images of humans who carry baggage. Thus, the transfer learning fastens the training procedure and improves the novel CNN model.

To adjust the hyperparameters related to the DenseNet method, the RMSProp optimizer is exploited in this study [27]. The RMSprop is an optimization algorithm for neural networks that uses the magnitude of recent gradients to normalize the rest of the gradients. It is similar to the standard Stochastic Gradient Descent (SGD) algorithm, but it uses a moving average of the squared gradient values to scale the learning rate. This function helps in preventing oscillations and divergence in the optimization process. The RMSprop is often used in Deep Learning techniques and is known to work well with Recurrent Neural Networks (RNNs). The RMSprop is an enhanced model of Adagrad, and its upgrading process is similar to that of the Adagrad optimizer. It calculates the exponential decay average of the squared gradient as given below:

(1) $$G_{t}=\beta G_{t-1}+\left(1-\beta \right)g_{t}⨀g_{t}=\left(1-\beta \right)\overset{t}{\underset{\tau =1}{\sum }}\beta^{t-\tau }g_{\tau }⨀g_{\tau },\;$$

In Equation (1), β denotes the decaying rate that is usually set as 0.9. In addition, the upgraded values of the parameter from the RMSprop are same as the Adagrad:

(2) $$\Delta \theta_{t}=-\frac{\alpha }{\sqrt{G_{t}+\varepsilon }}⨀g_{\tau }.\;$$

Furthermore, the simplified model of the Adagrad is given below:

(3) $$g_{t}^{\prime }=\frac{1}{\sqrt{G_{t}+\varepsilon }}⨀g_{\tau },\;$$

The upgraded value of the RMSprop is defined by Equation (4) as given below:

(4) $$\Delta \theta_{t}=-\alpha g_{t}^{\prime }.\;$$

Hence, the RMSprop is an enhanced method based on the gradient. To conduct the analysis, the rate of learning optimized approach is employed to enhance the training efficacy.

### 3.3. Prostate Cancer Classification

**Figure 2:**
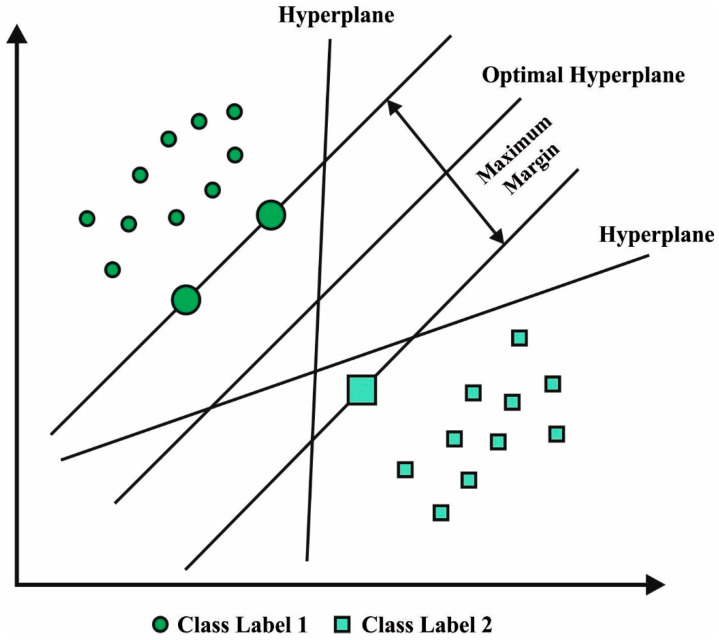
SVM hyperplane.

For PCa detection, the LS-SVM model is used. The LS-SVM is a well-organized ML algorithm that functions on the basis of statistical learning approach developed by Vapnik. The LSSVM algorithm effectively overcomes the higher-dimensional nonlinear and local minimal problems [28]. It can be established in a SVM method with two other features. Initially, inequality is constrained by the equality constraints after which it transmits two programming problems to the linear expression. These additional features accelerate the computational time of the LSSVM on the SVM method. Figure 2 demonstrates the structure of the SVM hyperplane.

The learning process of the LSSVM is shown below. $\left(x_{i},\;y_{i}\right),i=1,2,\;\ldots ,\;n$, where $x_{i}$ represents the $i^{th}$ predictor parameter and $y_{i}$ denotes the $i^{th}$ outcome parameter. The linear regression function is given below:

(5) $$f\left(x\right)=w^{T}g\left(x\right)+b\;$$

In Equation (5), w and b correspondingly determine the weight vector and the deviation.

(6) $$\min\;J\left(\omega ,\;\zeta \right)=\frac{1}{2}\omega^{T}\omega +\frac{1}{2}C\overset{m}{\underset{i=1}{\sum }}\zeta^{2}\;$$

In Equation (6), C and $\zeta_{i}$ correspondingly indicate the error penalty and the slack parameter. Based on Mercer’s condition, the linear kernel purpose is selected as shown below:

(7) $$K\left(x_{i},x_{j}\right)=\phi {\left(x_{i}\right)}^{T}\phi \left(x_{j}\right)\;$$

The final LSSVM approach is calculated based on the following expression:

(8) $$f\left(x\right)=\overset{m}{\underset{i=1}{\sum }}a_{i}K\left(x,x_{i}\right)+b\;$$

where $a_{i}$ indicates the Lagrangian multiplier.

To adjust the LS-SVM parameters, the current research work utilizes the AOA. Hashim et al. [29] proposed the AOA algorithm based on the Archimedes’ principle that is deemed to be the law of physics. This principle focuses on an object that is immersed, either fully or partly in a fluid. In general, there exists an upward force (termed ‘buoyancy’) exerted by the liquid on the body. This force corresponds to the weight of the fluid displaced by the body. In this work, immersed objects are deemed to be an individual population (i.e., solution candidates). This technique begins with the population initialization and, along with that, the position of each object is arbitrarily initiated inside the problem searching spaces. Then, their respective Fitness Function (FF) is evaluated. In the iteration model, the AOA upgrades the object density ($den_{i})$ and volume $\left(vol_{i}\right)$ of the *i^th^* object once the acceleration is upgraded based on the collision with a neighboring object. The position of all the objects ($O_{i}$) is defined as follows:

(9) $$O_{i}=l_{i}+rand\times \left(u_{i}-l_{i}\right),\;i=1,2,\;\ldots ,\;N\;$$

where $l_{i}$ and $u_{i}$ characterize the lower and upper limits of the $i^{th}$ object, and N represents the object amount.

(10) $$den_{i}=rand,\;vol_{i}=rand\;$$

Consider that rand represents a random number in the range of {0, 1}, the acceleration $\left(acc_{i}\right)$ of the *i^th^* objective is initialized as follows:

(11) $$acc_{i}=l_{i}+rand\times \left(u_{i}-l_{i}\right)\;$$

The initial FF is assessed, and the object with the optimal fitness is allotted as $x^{best},$ $den^{best},$ $vol^{best}$, and $acc^{best}$.

The updating methods of the $i^{th}$ object density and volume for the iteration t+1 are formulated as follows:

(12) $$den_{i}^{t+1}=den_{i}^{t}+rand\times \left(den^{best}-den_{i}^{t}\right)\;$$

(13) $$vol_{i}^{t+1}=vol_{i}^{t}+rand\times \left(vol^{best}-vol_{i}^{t}\right)\;$$

In these expressions, t denotes the current iteration and rand denotes a random number. At first, there exists a collision amongst the objects that try to accomplish the equilibrium state. This process is presented in the AOA through the Transfer Operator (TF) that assists in the transformation from the exploration stage to the exploitation stage:

(14) $$TF=\;\exp\;\left(\frac{t-t_{\max}}{t_{\max}}\right)\;$$

In this equation, t represents the iteration number and $t_{\max}$ denotes the maximal iteration count. Here, the TF values get increased gradually. The density reducing factor (d) helps the AOA to transfer from the global to the local search space:

(15) $$d^{t+1}=\;\exp\;\left(\frac{t_{\max}-t}{t_{\max}}\right)-\left(\frac{t}{t_{\max}}\right)\;$$

The values of $d^{t+1}$ reduce with time while, on the other hand, the proper allocation of these parameters helps in attaining a fine balance between the exploitation and exploration stages [30]. The exploration stage is signified as the collision among the objects, while the TF value is 0.5. The acceleration of the $i^{th}$ object at t+1 iterations is upgraded as follows:

(16) $$acc_{i}^{t+1}=\frac{den_{mr}+vol_{mr}\times acc_{mr}}{den_{i}^{t+1}\times vol_{i}^{t+1}}\;$$

Here, $acc_{mr}$ $den_{mr},$ and $vol_{mr}$, indicate the acceleration, density, and volume of the random material (mr). The exploitation phase in the AOA assumes that there is no collision among the objects. If TF > 0.5, there is no collision between the objects while an object’s acceleration is updated for the iteration t+1 using Equation (17):

(17) $$acc_{i}^{t+1}=\frac{den^{best}+vol^{best}\times acc^{best}}{den_{i}^{t+1}\times vol_{i}^{t+1}}\;$$

In Equation (17), $acc_{best}$ represents the acceleration of an object that is optimal with respect to fitness function. It is crucial to normalize the acceleration of each particle as it defines the step percentage that every particle might change and is given in the following expression:

(18) $$acc_{i-norm}^{t+1}=u\times \left(\frac{acc_{i}^{t+1}-\min\left(acc\right)}{\max\left(acc\right)-\min\left(acc\right)}\right)+l\;$$

In this expression, l and u characterize the normalization range while the values that are correspondingly allotted are 0.1 and 0.9. When an object is further away from the global optima, then the value of the acceleration would be higher. The exploration phase is conducted or else the exploitation phase is implemented.

The position of the $i^{th}$ particle gets upgraded in the exploration phase as follows:

(19) $$x_{i}^{t+1}=x_{i}^{t}+C_{1}\times rand\times acc_{i-norm}^{t+1}\times d\times \left(x_{rand}-x_{i}^{t}\right)\;$$

The location updating in the exploitation phase is formulated as follows:

(20) $$x_{i}^{t+1}=x_{i}^{t}+F\times C_{2}\times rand\times acc_{i-norm}^{t+1}\times d\times \left(T\times x_{best}-x_{i}^{t}\right)\;$$

*C_1_* is equal to 2, whereas *C_2_* is equal to 6. T increases with time and is directly proportional to the Transfer Operator. It is defined using the equation, *T* = $C_{3}$ × *TF*. $C_{3}$ shows a constant number, $x_{best}$ indicates the place of the optimum particle, and F represents the flag that is utilized to change the direction of particle movements. It can be determined using the following expression:

(21) $$F=\{\begin{array}{l} +1\;if\;P\leq 0.5\\ -1\;if\;P>0.5 \end{array}\;$$

where $P=2\times rand-C_{4}$. Finally, the *FF* values are evaluated at the upgraded location.

The AOA methodology processes a *FF* to realize the enhanced classification results. It describes a positive integer to achieve a good performance of the candidate results. In this case, a minimized classifier error rate is assumed to be the *FF* as expressed in Equation (22):

(22) $$fitness\left(x_{i}\right)=Classifier\;Error\;Rate\left(x_{i}\right)=\frac{number\;of\;misclassified\;samples}{Total\;number\;of\;samples}*100\;$$

## 4. Results and Discussion

**Figure 3:**
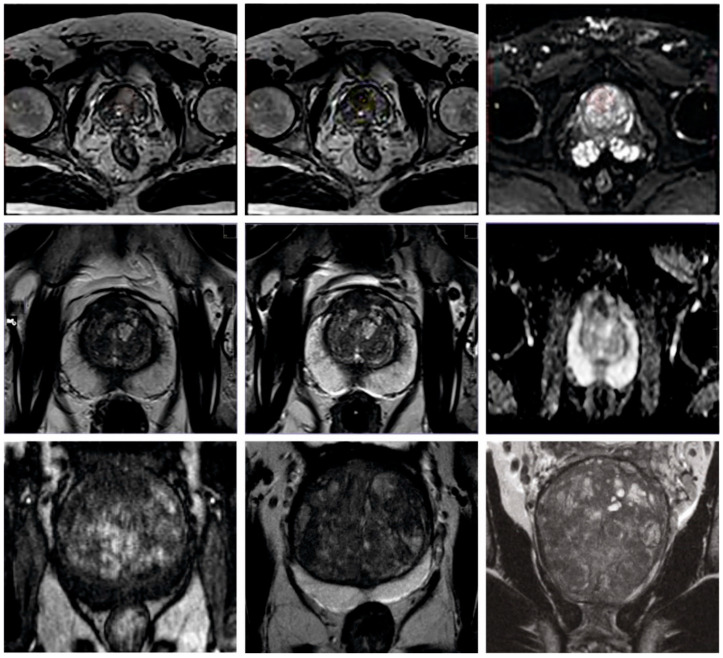
Sample images.

The proposed model was simulated using the Python 3.6.5 tool on a PC configured with the following specifications: i5-8600k, GeForce 1050Ti 4GB, 16GB RAM, 250GB SSD, and 1TB HDD. The parameter settings are learning rate: 0.01, dropout: 0.5, batch size: 5, epoch count: 50, and activation: ReLU. The current section discusses the PCa classification results achieved by the proposed AOADLB-P2C model. The model was tested on a dataset comprising 400 samples under two classes, as defined in Table 1. Figure 3 depicts some of the sample images used in this study. 

**Figure 4:**
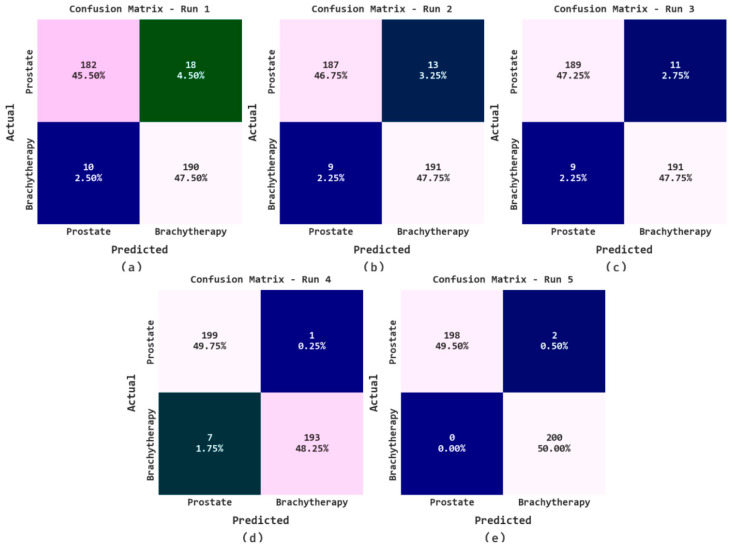
Confusion matrices of the proposed AOADLB-P2C system: (**a**) Run-1, (**b**) Run-2, (**c**) Run-3, (**d**) Run-4, and (**e**) Run-5.

The confusion matrices generated by the proposed AOADLB-P2C model during different runs of execution are displayed in Figure 4. The figure highlights that the proposed AOADLB-P2C method recognized the images under the two classes accurately.

**Figure 5:**
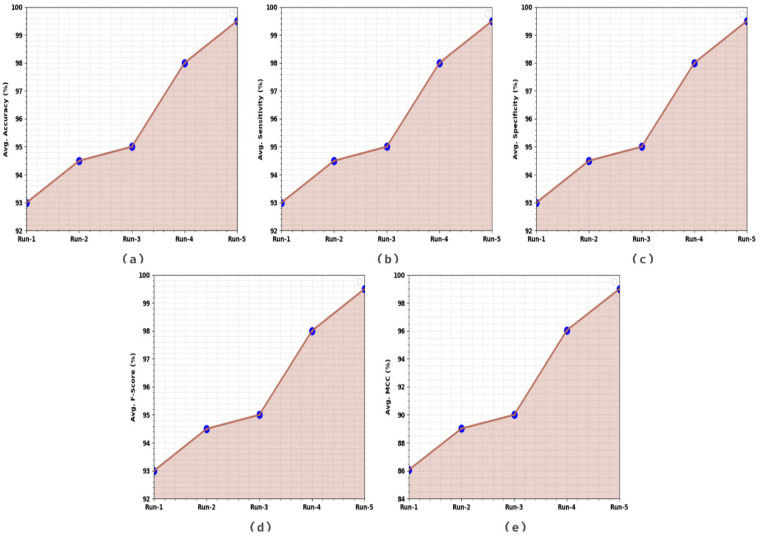
Average analysis of the AOADLB-P2C system: (**a**) Run-1, (**b**) Run-2, (**c**) Run-3, (**d**) Run-4, and (**e**) Run-5.

Table 2 and Figure 5 show the overall classification outcomes achieved by the proposed AOADLB-P2C model under five runs. The outcomes imply that the presented AOADLB-P2C model achieves enhanced outcomes under each run. For example, on run-1, the AOADLB-P2C model acquires an average $accu_{bal}$ of 93%, $sens_{y}$ of 93%, $spec_{y}$ of 93%, $F_{score}$ of 93%, and a MCC of 86.07%. Meanwhile, on run-3, the proposed AOADLB-P2C method obtains an average $accu_{bal}$ of 95%, $sens_{y}$ of 95%, $spec_{y}$ of 95%, $F_{score}$ of 95%, and a MCC of 90%. Eventually, on run-5, the proposed AOADLB-P2C approach acquires an average $accu_{bal}$ of 99.50%, $sens_{y}$ of 99.50%, $spec_{y}$ of 99.50%, $F_{score}$ of 99.50%, and a MCC of 99%.

**Figure 6:**
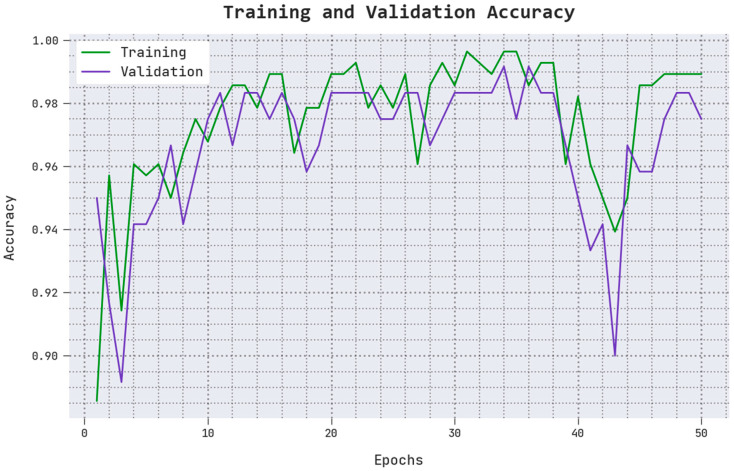
TACC and VACC analytical results of the AOADLB-P2C system.

The TACC and VACC values achieved by the proposed AOADLB-P2C technique on PCa performance are shown in Figure 6. The figure infers that the proposed AOADLB-P2C approach exhibits an improved performance with increased TACC and VACC values. It is also noted that the AOADLB-P2C algorithm reaches the maximum TACC outcomes.

**Figure 7:**
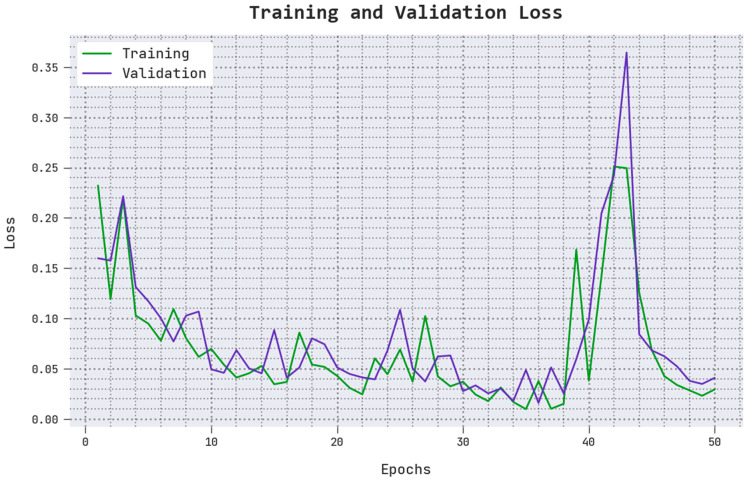
TLS and VLS analytical results of the proposed AOADLB-P2C system.

The TLS and VLS values accomplished by the proposed AOADLB-P2C technique on PCa performance are portrayed in Figure 7. The figure shows that the AOADLB-P2C approach exhibits a superior performance with minimal TLS and VLS values. It is to be noted that the proposed AOADLB-P2C methodology produces the lowest VLS outcomes.

**Figure 8:**
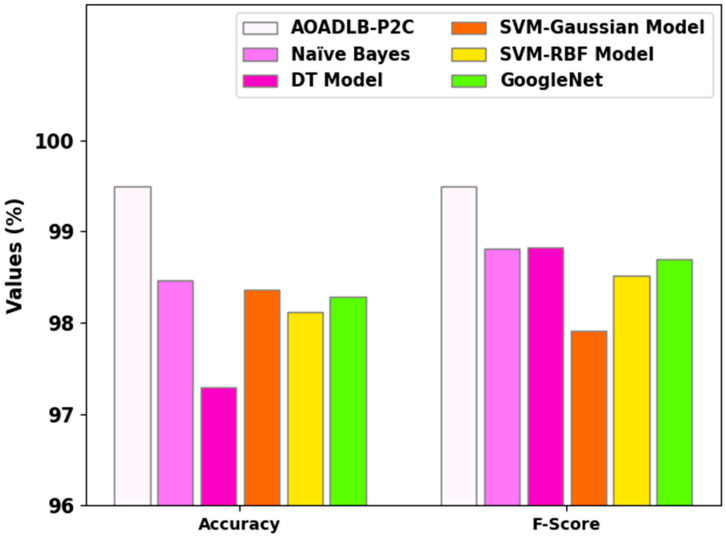
$Accu_{y}$ and $F_{score}$ analytical results of the AOADLB-P2C system and other recent approaches.

In Table 3, the outcomes of the comprehensive comparative study achieved by the proposed AOADLB-P2C method and other recent models are given [10]. Figure 8 reports the results from the comparative $accu_{y}$ and $F_{score}$ inspection, attained by the proposed AOADLB-P2C method and other techniques. The results specify that the AOADLB-P2C method accomplish superior results over other models. In terms of $accu_{y}$, the AOADLB-P2C model obtains a maximum $accu_{y}$ of 99.50% while the other models, including the NB, DT, SVM-Gaussian, SVM-RBF, and GoogleNet, obtain lower $accu_{y}$ values of 98.46%, 97.29%, 98.36%, 98.12%, and 98.28% respectively. In addition, based on the $F_{score}$, the AOADLB-P2C technique achieves the highest $F_{score}$ of 99.50%, while the NB, DT, SVM-Gaussian, SVM-RBF, and GoogleNet approaches achieve lower $F_{score}$ values of 98.81%, 98.83%, 97.91%, 98.52%, and 98.69%, respectively.

**Figure 9:**
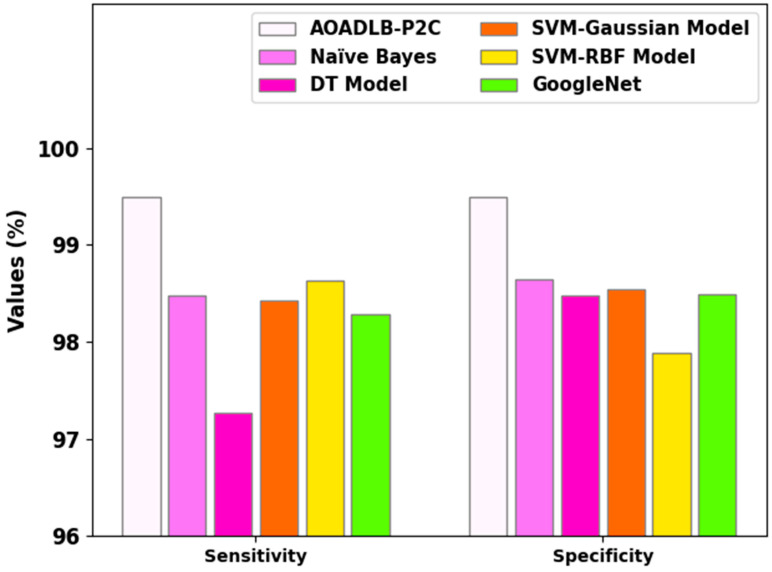
$Sens_{y}$ and $Spec_{y}$ analytical results of the proposed AOADLB-P2C system and other recent approaches.

Figure 9 illustrate the $sens_{y}$ and $spec_{y}$ analytical outcomes accomplished by the proposed AOADLB-P2C methodology and other existing approaches. The outcomes show that the AOADLB-P2C approach exhibits a superior performance over other techniques. In terms of $sens_{y}$, the proposed AOADLB-P2C methodology attains a maximum $sens_{y}$ of 99.50%, while the NB, DT, SVM-Gaussian, SVM-RBF, and GoogleNet methods attain lower $sens_{y}$ values of 98.47%, 97.26%, 98.43%, 98.63%, and 98.28%, respectively. Likewise, in terms of $spec_{y}$, the AOADLB-P2C technique attains a maximum $spec_{y}$ of 99.50%, whereas the NB, DT, SVM-Gaussian, SVM-RBF, and GoogleNet approaches attain lower $spec_{y}$ values of 98.641%, 98.47%, 98.54%, 97.89%, and 98.49%, respectively.

These results confirm the improved outcomes of the proposed AOADLB-P2C model on prostate cancer classification process. The enhanced performance of the proposed model is due to the inclusion of AMF-based pre-processing, DenseNet-161-based feature extraction, RMSProp optimizer, LS-SVM classification, and AOA-based hyperparameter tuning. Therefore, the proposed model can be employed for accurate PCa detection and classification using MRI images.

## 5. Conclusions

In this research work, the authors introduced a new AOADLB-P2C model for PCa diagnosis using MRI images. Initially, the AOADLB-P2C model pre-processes MRI images in two stages using AMF-based noise removal and contrast enhancement. Moreover, the RMSProp optimizer is applied with the DenseNet-161 model for the purpose of feature extraction. Finally, the presented AOADLB-P2C model classifies PCa using the AOA with the LS-SVM method. The presented AOADLB-P2C method was experimentally evaluated using a benchmark MRI dataset. The comparative simulation values confirmed the improved performance of the proposed AOADLB-P2C methodology over other recent methodologies, with a maximum accuracy of 99.50%. Hence, the presented AOADLB-P2C method can be employed for PCa classification. In the future, the efficiency of the AOADLB-P2C model can be improved using ensemble fusion models.

## Figures and Tables

**Table 1:** Dataset details.

Class	No. of Instances (Balanced)
Prostate	200
Brachytherapy	200
Total Number of Instances	400

**Table 2:** Analytical results of the AOADLB-P2C system under distinct measures and runs.

Class	Accuracy_bal_	Sensitivity	Specificity	F-Score	MCC
Run-1					
Prostate	91.00	91.00	95.00	92.86	86.07
Brachytherapy	95.00	95.00	91.00	93.14	86.07
Average	93.00	93.00	93.00	93.00	86.07
Run-2					
Prostate	93.50	93.50	95.50	94.44	89.02
Brachytherapy	95.50	95.50	93.50	94.55	89.02
Average	94.50	94.50	94.50	94.50	89.02
Run-3					
Prostate	94.50	94.50	95.50	94.97	90.00
Brachytherapy	95.50	95.50	94.50	95.02	90.00
Average	95.00	95.00	95.00	95.00	90.00
Run-4					
Prostate	99.50	99.50	96.50	98.03	96.04
Brachytherapy	96.50	96.50	99.50	97.97	96.04
Average	98.00	98.00	98.00	98.00	96.04
Run-5					
Prostate	99.00	99.00	100.00	99.50	99.00
Brachytherapy	100.00	100.00	99.00	99.50	99.00
Average	99.50	99.50	99.50	99.50	99.00

**Table 3:** Comparative analytical results of the proposed AOADLB-P2C system and other recent approaches.

Methods	Accuracy	Sensitivity	Specificity	F-Score
AOADLB-P2C	99.50	99.50	99.50	99.50
Naïve Bayes	98.46	98.47	98.64	98.81
DT Model	97.29	97.26	98.47	98.83
SVM-Gaussian Model	98.36	98.43	98.54	97.91
SVM-RBF Model	98.12	98.63	97.89	98.52
GoogleNet	98.28	98.28	98.49	98.69

## Data Availability

Data sharing is not applicable to this article as no datasets were generated during the current study.
